# Rabies outbreak in black-backed jackals (*Canis mesomelas*), South Africa, 2016

**DOI:** 10.1017/S0950268821002685

**Published:** 2022-01-07

**Authors:** E. Ngoepe, J. G. Chirima, D. Mohale, K. Mogano, T. Suzuki, K. Makita, C. T. Sabeta

**Affiliations:** 1 Agricultural Research Council, Onderstepoort Veterinary Institute, Onderstepoort, 0110, Pretoria, Gauteng, South Africa; 2 GeoInformation Science Division, Agricultural Research Council, Institute for Soil, Climate and Water 600, Belvedere St, Arcadia, Pretoria, Gauteng, South Africa; 3 Department of Geography, Geoinformatics and Meteorology, Centre for Geoinformation Science, University of Pretoria, Pretoria, Gauteng, South Africa; 4 Department of Veterinary Medicine, School of Veterinary Medicine, Rakuno Gakuen University, 582 Bunkyodai Midorimachi, Ebetsu, Hokkaido, 069-8501, Japan; 5 Veterinary Tropical Diseases, University of Pretoria, Onderstepoort, 0110, Pretoria, Gauteng, South Africa

**Keywords:** Black-backed jackal, *C. mesomelas*, cross-species transmission, Lyssaviruses, rabies, South Africa

## Abstract

Rabies, a fatal and vaccine-preventable disease, is endemic throughout Africa. In 2016, a rabies outbreak occurred in black-backed jackals (*Canis mesomelas*) along the western boundary of Gauteng Province, South Africa. We investigated the possible drivers of the 2016 outbreak and established its origin. Using spatio-temporal locations of cases, we applied logistic regression and Geographic Information System techniques to investigate environmental covariates driving occurrences of emerging rabies cases in Gauteng Province. About 53.8% of laboratory-confirmed lyssaviruses in Gauteng Province in 2016 originated from jackals. Phylogenetic trees reconstructed from a partial region of the glycoprotein gene of these and historical rabies viruses (RABVs) demonstrated the lyssaviruses to be of canid origin with 97.7% nucleotide sequence similarity. The major cluster comprised jackal RABVs from the 2012 KwaZulu/Natal outbreak and the 2016 outbreak in Gauteng Province. The second cluster was composed of both jackal and dog RABVs. Both clusters correlated with independent RABV introductions into Gauteng by dogs and jackals, respectively. This study demonstrated an expansion of a jackal rabies cycle from north-west Province into Gauteng Province during the 2016 dry period, as jackals ranged widely in search for food resources leading to increased jackal-dog interactions, reminiscent of the intricate links of domestic and wildlife rabies cycles in South Africa.

## Introduction

Rabies virus (RABV), is a prototype species of the *Lyssavirus* genus (*Rhabdoviridae* family, order *Mononegavirales*)**,** and currently consists of 17 viral species [1]. In addition, Kotalahti bat lyssavirus (KBLV), a putative species discovered in a Brandt's bat (*Myotis brandtii*) in Finland, is still awaiting formal classification [2]. All lyssaviruses are capable of causing an encephalitic disease in all warm-blooded vertebrates. The domestic dog (*Canis familiaris*) is the primary vector species for rabies and is responsible for at least 59 000 human deaths annually [3], with the majority (⩾95%) of these deaths occurring in the low endemic countries of Africa and Asia [4, 5]. Rabies is a neglected and re-emerging zoonosis in specific regions of Africa and Asia, and poses a significant public health problem in these geographical areas [6, 7]. In the rabies endemic regions of Africa and Asia, the disease is highly under-reported primarily due to inadequate diagnostic facilities and the long distances specimens have to be transported to reach the diagnostic facilities [5, 6]. Unlike in Africa, in Europe and North America, rabies was eliminated through parenteral dog vaccination [8, 9]. However, the disease is still endemic in some wildlife species [such as red foxes (*Vulpes vulpes*) and raccoon dogs (*Nyctereutes procyonoides*)] in some European countries [8, 9].

In South Africa**,** rabies cycles are maintained by both domestic and wildlife host species in specific geographical regions of the country. Cross-species transmission events (CSTEs), between domestic dogs and two wildlife host species, the black-backed jackals and the bat-eared fox *Otocyon megalotis*, facilitate the ease of exchange of canid RABVs in the northern and western regions of South Africa [10–16]. On the Highveld plateau of the Free State province of South Africa, mongoose rabies, believed to be indigenous to the sub-region, and introduced over 200 years ago, is maintained by a diverse group of members of the *Herpestidae* family [17]. The presence of two independent rabies cycles of the canid and mongoose rabies biotypes is unique and complicates rabies epidemiology in the country [16]. However, mongoose rabies does not appear to pose much of a public health threat compared to canid rabies [17]. Recent trends analyses from the Onderstepoort Records seem to suggest that the aardwolf (*Proteles cristata*), a small insectivorous mammalian carnivore native to East and Southern Africa, could potentially maintain canid rabies (Onderstepoort rabies records).

RABVs originating from dogs and jackals are very closely related and highlight CSTEs of RABV variants between domestic (dogs) and wildlife host species black-backed jackals in commercial farming areas in central and north-western Limpopo [15, 16]. Rabies cycles are evidently maintained by both domestic dogs and black-backed jackals in specific geographical areas. The cycles are more pronounced in Zimbabwe**,** in communal areas adjacent to nature reserves and commercial farming areas [13, 14, 18–20]. Jackal rabies (canid rabies) which originated from dogs [15, 16, 21] has been maintained in jackal species consistently for at least 50 years in northern South Africa. Similarly, in Zimbabwe, rabies epidemics in jackal species are thought to have been initiated through similar spillover events and once they were established, the epidemics were maintained by black-backed jackals [14]. Incidentally, black-backed jackals were believed incapable of maintaining continuous rabies cycles because of their low population densities when compared to domestic dogs [14, 22, 23].

In South Africa, there are two testing laboratories for animal rabies, one the OIE Rabies Reference Laboratory at Onderstepoort**,** in Gauteng Province, and the Allerton Provincial Laboratory in Pietermaritzburg KwaZulu/Natal (KZN). Firstly, we wanted to establish rabies trends in this wildlife carnivore species in Gauteng Province and secondly, to antigenically and genetically characterise RABVs originating from the 2016 jackal rabies outbreak. Thirdly, we wanted to assess the relations between spatial and temporal rabies cases with environmental explanatory variables as a way to identify risk factors in historically unrecorded areas, and finally, we wanted to reconstruct phylogenetic relationships of the RABVs from the 2016 outbreak together with previously characterised viruses from other wildlife and domestic host species.

## Materials and methods

### Specimens

**Fig. 1.**
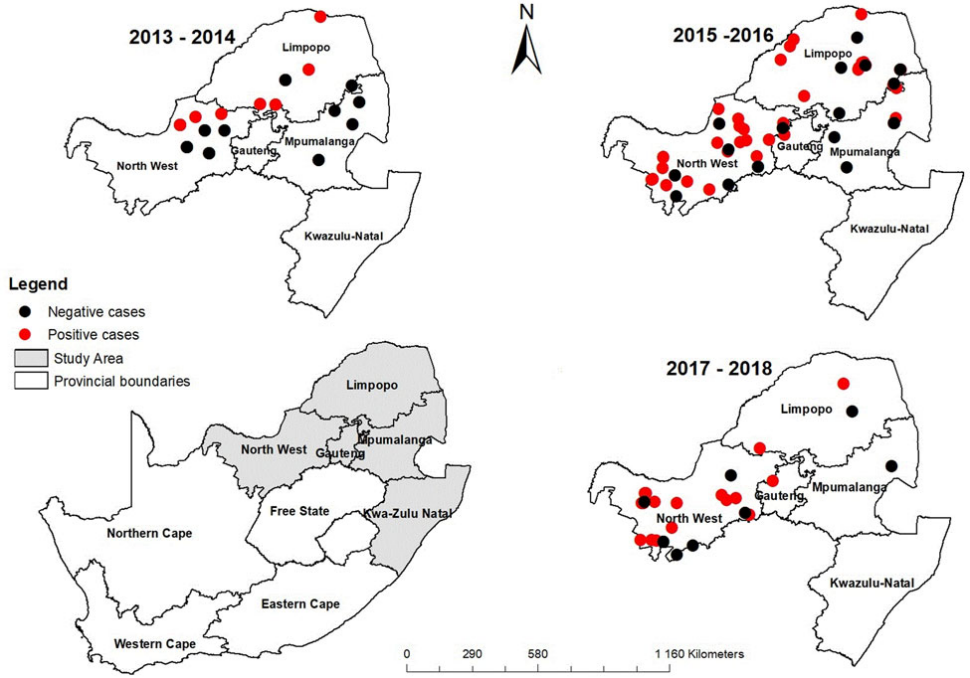
Jackal cases for the periods 2013–2014, 2015–2016 and 2017.

Specimens from a variety of central nervous system tissues, mostly in the form of intact brain tissues obtained in 2016 through the national rabies surveillance programme, were submitted to the Agricultural Research Council-Onderstepoort Veterinary Institute (ARC-OVI) for lyssavirus diagnosis (Fig. 1, Table 1). In terms of the Animal Disease Act of the Republic of South Africa (Act 35 of 1984), rabies is a controlled disease. By law, a controlled animal disease is a disease that must be reported to the nearest state veterinarian and control measures must be prescribed for its control. Hence, any procedures for collecting specimens including euthanising animals to enable laboratory diagnosis of the specimens, are vested in the state veterinarian through the Act.

**Table 1.** Epidemiological information of RABVs genetically characterised in this study

Virus no.	Lab. reference no.	Locality of origin	Province	Host species of origin	Co-ordinates (longitude-latitude)	Genbank Accession Numbers
1	45/94	Warmbad	Limpopo	*Canis mesomelas*	27.44–24.52	EF686064
2	504/96	Warmbad	Limpopo	*Canis mesomelas*	27.44–24.56	AF177108
3	306/98	Warmbad	Limpopo	*Canis mesomelas*	28.07–24.51	AF177105
4	418/99	Polokwane	Limpopo	*Canis mesomelas*	29.50–23.45	EF686070
5	549/99	Polokwane	Limpopo	*Canis mesomelas*	29.24–24.04	EF686069
6	557/99	Polokwane	Limpopo	*Canis mesomelas*	29.24–24.05	EF686054
7	669/99	Polokwane	Limpopo	*Canis mesomelas*	29.27–23.47	AF303062
8	673/99	Mokopane	Limpopo	*Canis mesomelas*	28.36–22.43	AF303061
9	717/99	Polokwane	Limpopo	*Canis mesomelas*	29.29–23.42	AF303064
10	1004/99	Polokwane	Limpopo	*Canis familiaris*	29.14–23.43	EF686052
11	433/01	Lephalale	Limpopo	*Canis mesomelas*	27.23–23.39	EF686084
12	307/02	Lephalale	Limpopo	*Canis mesomelas*	27.37–23.29	EF686081
13	629/02	Lephalale	Limpopo	*Canis mesomelas*	27.33–23.23	EF686083
14	543/03	Lephalale	Limpopo	*Canis mesomelas*	27.11–23.48	EF686088
15	588/07	Pretoria (Paardefontein)	Gauteng	*Canis familiaris*	29.21–27.46	JF327503
16	598/07	Pretoria (Paardefontein)	Gauteng	*Canis familiaris*	28.23–25.35	JF327500
17	655/07	Pretoria (Plot 31 Kallagte 122)	Gauteng	*Canis mesomelas*	25.25–28.22	JF327501
18	894/07	Pretoria	Gauteng	*Canis mesomelas*	25.17–28.38	JF327502
19	115/11	Randfontein	Gauteng	*Canis familiaris*	27.70–26.17	JN227482
20	157/11	Germiston	Gauteng	*Canis familiaris*	28.16–26.21	JN227483
21	109/16	Pretoria	Gauteng	*Felis catus*	28.22–25.74	MW413399
22	326/16	Cullinan, Pretoria	Gauteng	*Mellivora capensis*	28.52–25.67	MW413409
23	380/16	Randfontein	Gauteng	*Canis mesomelas*	27.70–26.17	MW413410
24	404/16	Tswaing	Gauteng	*Canis mesomelas*	28.10–25.24	MW413411
25	409/16	Randfontein	Gauteng	*Canis mesomelas*	26.07–27.73	MW413412
26	422/16	Elandsfontein	Gauteng	*Bovis taurus*	26.36–28.19	MW413413
27	428/16	Renasterpruit	Gauteng	*Canis mesomelas*	26.38–26.89	MW413414
28	466/16	Paoloni, Randfontein	Gauteng	*Canis mesomelas*	27.70–26.17	MW413400
29	468/16	Tweefontein	Gauteng	*Bovis taurus*	28.34–26.03	MW413401
30	486/16	Randfontein	Gauteng	*Canis mesomelas*	27.70–26.17	MW413402
31	489/16	Tweefontein	Gauteng	*Bovis taurus*	28.34–26.03	MW413403
32	490/16	Haartebeesfontein	Gauteng	*Canis mesomelas*	28.29–25.27	MW413404
33	527/16	Randfontein	Gauteng	*Bovis taurus*	27.70–26.17	MW413405
34	541/16	Kroomdraai	Gauteng	*Canis mesomelas*	28.33–25.79	MW413406
35	542/16	Kroomdraai	Gauteng	*Canis mesomelas*	28.33–25.79	MW413407
36	551/16	Swartruggens	North West	*Canis mesomelas*	26.59–26.02	MW413408
37	454/17	Rietkuil, Potchefstroom	North West	*Canis mesomelas*	26.14–27.12	MT454646
38	460/17	Niekerkrus, Ganyesa	North West	*Canis mesomelas*	24.27–26.16	MT454647
39	466/17	Rooipoort, Potchefstroom	North West	*Canis mesomelas*	26.57–26.35	MT454648
40	474/16	Matlhako, Taung	North West	*Canis mesomelas*	24.12–27.49	MT454649
41	480/17	Blouboskuil, Mamusa	North West	*Canis mesomelas*	24.57–27.51	MT454651
42	483/17	Klipplaatdrift, Ventersdorp	North West	*Canis mesomelas*	26.45–26.25	MT454652
43	502/17	Kroomdraai, Potchefstroom	North West	*Canis mesomelas*	26.55–26.51	MT454653
44	503/17	Rooipoort, Potchefstroom	North West	*Canis mesomelas*	26.57–26.35	MT454654
45	125/15	Sun city	North West	*Canis familiaris*	Not provided	MT454631
46	471/15	Kwarrefontein	North West	*Canis familiaris*	26.07–26.37	MT454634
47	682/15	Madidi	North West	*Canis familiaris*	Not provided	MT454635
48	516/16	Weltevreden	North West	*Canis familiaris*	27.09–26.04	MT454636
49	583/16	Saalsport	North West	*Canis familiaris*	27.12–25.10	MT454637
50	635/16	Skuilkloof	North West	*Canis familiaris*	Not provided	MT454639
51	690/16	Palmietfontein	North West	*Canis familiaris*	26.39–26.34	MT454641
52	269/17	Schwaing	North West	*Canis familiaris*	24.49–26.36	MT454643
53	400/17	Vryburg	North West	*Canis familiaris*	24.23–26.40	MT454645
54	172/15	Lerato	North West	*Canis mesomelas*	Not provided	Awaiting accession
55	520/14	Lerato	North West	*Canis mesomelas*	Not provided	Awaiting accession
56	491/14	Lerato	North West	*Canis mesomelas*	Not provided	Awaiting accession
57	380/14	Vlakte Swartruggens	North West	*Canis mesomelas*	Not provided	Awaiting accession
58	15/183	Not provided	Kwa-Zulu Natal	*Canis familiaris*	Not provided	KY681392
59	15/41	Not provided	Kwa-Zulu Natal	*Canis mesomelas*	Not provided	KY681378
60	15/96	Not provided	Kwa-Zulu Natal	*Canis mesomelas*	Not provided	KY681381
61	16/010	Not provided	Kwa-Zulu Natal	*Canis mesomelas*	Not provided	KY681432
62	12/680	Not provided	Kwa-Zulu Natal	*Canis mesomelas*	Not provided	KY681374
63	12/830	Not provided	Kwa-Zulu Natal	*Canis mesomelas*	Not provided	KY681371
64	15/22	Not provided	Kwa-Zulu Natal	*Canis mesomelas*	Not provided	KY681377
65	15/74	Not provided	Kwa-Zulu Natal	*Canis mesomelas*	Not provided	KY681380
66	15/595	Not provided	Kwa-Zulu Natal	*Canis mesomelas*	Not provided	KY681407
67	15/19	Not provided	Kwa-Zulu Natal	*Canis mesomelas*	Not provided	KY681376
68	12/616	Not provided	Kwa-Zulu Natal	*Canis mesomelas*	Not provided	KY681365
69	15/187	Not provided	Kwa-Zulu Natal	*Canis mesomelas*	Not provided	KY681394
70	15/158	Not provided	Kwa-Zuprovidedlu Natal	*Canis mesomelas*	Not provided	KY681387
71	12/621	Not provided	Kwa-Zulu Natal	*Canis mesomelas*	Not provided	KY681373
72	15/279	Not provided	Kwa-Zulu Natal	*Canis mesomelas*	Not provided	KY681397
73	15/110	Not applicable	Kwa-Zulu Natal	*Canis mesomelas*	Not applicable	KY681382
74	15/53	Not applicable	Kwa-Zulu Natal	*Canis mesomelas*	Not applicable	KY681379
75	12/407	Not applicable	Kwa-Zulu Natal	*Canis familiaris*	Not applicable	KY681366
76	15/286	Not applicable	Kwa-Zulu Natal	*Canis mesomelas*	Not applicable	KY681398
77	15/157	Not applicable	Kwa-Zulu Natal	*Canis mesomelas*	Not applicable	KY681386
78	15/563	Not applicable	Kwa-Zulu Natal	*Canis familiaris*	Not applicable	KY681406
79	16/069	Not applicable	Kwa-Zulu Natal	*Canis mesomelas*	Not applicable	KY681414
80	16/102	Not applicable	Kwa-Zulu Natal	*Canis mesomelas*	Not applicable	KY681416
81	15/184	Not applicable	Kwa-Zulu Natal	*Canis mesomelas*	Not applicable	KY681393

A total of 190 brain specimens originating from both domestic and wildlife host species were received. These included specimens from domestic dogs (*Canis familiaris*) (*n* = 71, 37.4%), bovines (*Bos taurus*) (*n* = 11; 5.8%), domestic cats (*Felis catus*) (*n* = 31; 16.3%), mongooses and other *Herpestidae* species (*n* = 17; 8.9%), black**-**backed jackal species (*n* = 39; 20.5%), unidentified bat species (Chiroptera) (*n* = 2; 1.1%), ovines (*Ovis aries)* (*n* = 2; 1.1%)**,** equines (*Equus caballus*) (3; 1.6%) and baboons (*Papio ursinus*) (*n* = 2; 1.1%). The remainder (*n* = 12) were from honey badgers (*Mellivora capensis*) (*n* = 2), mice (*Mus musculus*) (*n* = 3), one each from a porcine (*Suidae*), rabbit (*Oryctolagus cuniculus*), rat (*Rattus rattus*), kudu (*Tragelaphus strepsiceros*), warthog (*Phacochoerus africanus*), impala (*Aepyceros melampus*) and sable antelope (*Hippotragus niger*).

### Primary rabies diagnosis

All the specimens including a positive and negative control**,** were tested using the direct fluorescent antibody (DFA) test described previously [24]. After staining, the slides were examined under ultra-violet fluorescence (Zeiss, Germany). The results of the DFA were recorded as positive [on a scale from + 1 (lyssavirus antigen present in 25% of the fields of the examined smear) to + 4 (lyssavirus antigen present in 100% of fields of the examined smear)] or negative (–).

### Antigenic typing

Antigenic typing of the lyssaviruses was performed using a panel of 16 murine anti-lyssavirus nucleocapsid monoclonal antibodies (anti-N Mabs), a donation kindly provided by Dr Christine-Fehlner Gardiner (Centre of Expertise for Rabies, Canadian Food Inspection Agency, Canada)**.** The 16 anti-N Mab panel, included 14 anti-RABV nucleoprotein mAbs, an anti-human adenovirus type-5 mAb (1C5) as a negative control and positive control (38HF2). The reactivity patterns generated from this Mab panel are capable of discriminating the different southern African *Lyssavirus* species and antigenic variants [25] (Table 2). Brain smears were prepared on Teflon-coated 5 mm well slides (Thermo Fisher Scientific, USA) and were acetone**-**fixed as per the DFA, described under primary rabies diagnosis. After staining with FITC-conjugated goat anti-mouse IgG antibody (Sigma-Aldrich, USA), the slides were blot-dried, an aqueous mounting medium added and then examined under ultra-violet microscopy (Carl Zeiss, Germany). The reactivity for each mAb was recorded as positive (on a scale from 1–3) or negative (−) for each lyssavirus to generate the overall staining pattern.

**Table 2.** The reactivity patterns of the rabies viruses analysed

	Canid 1 rabies biotype	Canid 2 rabies biotype	Mongoose rabies virus	Lagos bat virus	Mokola virus	Duvenhage virus	jackal 83/16	Dog 286/15
1C5	–	–	–	–	–	–	–	–
26AB7	+++	+++	Var	–	–	–	+++	+++
26BE2	+++	+++	Var	–	–	–	+++	+++
32GD12	+++	–	Var	–	–	–	+++	+++
38HF2	+++	+++	+++	+++	+++	+++	+++	+++
M612	–	–	–	+++	–	–	–	–
M837	–	–	–	–	–	+++	–	–
M850	–	–	Var	–	–	+++	–	–
M853	+++	+++	–	–	–	+++	+++	+++
M1001	––	–	–	–	+++	–	–	–
M1335	–	–	Var	–	var	–	–	–
M1386	–	–	+++	–	–	–	–	–
M1400	–	–	Var	–	–	–	–	–
M1407	++	++	Var	–	–	–	++	++
M1412	++	++	Var	–	–	–	++	++
M1494	–	–	Var	–	–	+++	–	–
	85.7%	14.3%					Canid	Canid

Key: Var, Some isolates within the species react with the Mab and others do not; +++, Reactivity observed; –, No reactivity observed.

### Total RNA extractions, reverse transcription PCR (RT-PCR), nucleotide sequencing and phylogenetic reconstruction

Total RNAs were extracted from approximately 100 ng of brain-infected tissues using TriReagent^®^ (Sigma Aldrich, USA)**.** Complementary DNA **(**cDNA**)** was synthesised using standard guidelines [26, 27] and the highly variable glycoprotein and G-L intergenic region of each virus was sequenced (Table 1). The nucleotide sequences for the forward and reverse primers used in the RT-PCR, G(+) and L(−), are _4665_^5^GAC TTG GGT CTC CCG AAC TGG GG^3^_4687_ & _5520_^5^CAA AGG AGA GTT GAG ATT GTA GTC^3^_5543_, respectively. The annealing positions and polarity are designated according to the Pasteur Virus genome [26, 27]. The primers were synthesised by Inqaba Biotechnical Industries (Pty) Ltd (Pretoria, South Africa).

In brief, approximately 1 μg of total viral RNA and G(+) primer were annealed at 70 °C. Thereafter, the RNAs were reverse-transcribed in a 20-μl reaction mixture, that consisted of 200 U of Superscript III Reverse Transcriptase (Invitrogen, USA), 5× SS III reaction buffer, 20 mm dNTP mix (Takara, Japan), 40 U RNasin RNase inhibitor of murine origin (New England Biolabs, USA)**,** and ultrapure diethyl-pyrocarbonate (DEPC)-treated water (Invitrogen, USA). The reaction mixture was incubated at 50 °C for 50 min and then the cDNA mixture inactivated at 85 °C for 10 min.

Two microliters of cDNA were used as template in a 50 μl-PCR reaction comprising 20 pmoles each of the G(+) and L(−) primers [26, 27], 20 mm dNTP mixture, 1.5 mm MgCl_2_, 5 ×  Taq DNA polymerase reaction buffer and 1.25 U of Takara Taq DNA polymerase (Takara, Japan). The reaction mixture was thermal-cycled starting with an initial denaturation at 94 °C for 2 min, then followed by 30 cycles of denaturation, 94 °C for 50 s, annealing 42 °C for 90s, extension 72 °C for 2 min and a final extension for 10 min. The PCR amplicons (5-μl volumes) were separated on 1% ethidium bromide-stained gels. Amplicons (45-μl volumes) were then purified using spin-columns.

The purified amplicons were sequenced bidirectionally using the reverse and forward primers and the edited nucleotide sequences aligned in ClustalW [28]. Thereafter**,** phylogenetic trees were reconstructed using both the Neighbour-Joining (NJ) method and Maximum Likelihood, ML [29] algorithms. Duplicate nucleotide sequences were removed from the analysis. The topology of the trees was validated with 1000 bootstrap replicates [30].

### Spatio-temporal variation of rabies cases in Gauteng

To assess for spatial heterogeneity and temporal dependence of cases in Gauteng, we extracted spatially explicit environmental covariates as predictor variables: Normalised Difference Vegetation Index (NDVI) (surrogate for forage productivity and forage retention), rainfall and temperature. Then, we applied logistic regression [31] to relate these candidate predictors to rabies cases for the period 2009–2018.

### Rainfall and temperature data

ArcMap 10.6 (Esri, Redlands, CA, USA) was used for all the spatial techniques described. First, a shapefile of all South African provinces was imported into, projected into Universal Transverse Mercator 1984, and then clipped to select only the area of interest, the Gauteng Province. Second, an Excel sheet of geo-locations of confirmed rabies positive cases obtained from the ARC-OVI was converted to a shapefile and overlaid on the Gauteng province shapefile to identify specific areas in the province with rabies cases (Fig. 1). Then, environmental covariates were derived for the Gauteng Province for specific locations of confirmed rabies cases and non-rabies cases.

Temperature surface maps were created for the following periods**:** pre-dry conditions (2009 up to 2013/2014), during dry conditions (2015–2016), and post-dry conditions (post 2017–2018). Weather station data available from the Agricultural Research Council (ARC) of South Africa and the South African Weather Services (SAWS) were obtained. Thin plate splines and regression interpolation of the weather station data as a function of latitude, longitude and elevation, was used to create climate surfaces. The mean annual temperature surface and dry season mean temperatures surface were indicative of possible heat stress and quick drying of forage.

An inverse distance weight method was applied to create a smooth surface trend on monthly rainfall using data available from the ARC and additional records from SAWS. The above rainfall surface was used to extract values of annual mean rainfall, dry season mean at a 1 km resolution. A long-term mean rainfall figure for each calendar month was extracted per weather station and a trend surface created from these monthly data using the inverse distance weight interpolation method. A spatial filter was applied to the interpolated surface to create a smooth trend surface. Then, regression analysis was applied to relate the differences between the rainfall values at a specific station and the value given by the trend surface to topographic indices such as terrain ruggedness, rain shadow, aspect, etc. The relationship and trend surface for each month were used to model a mean rainfall surface from spatial topographic indices. To obtain an annual total mean rainfall surface, the individual monthly surfaces were summed. Long-term average rainfall and temperature (monthly surfaces) for the period prior to dry conditions (2009 up to 2013/2014), during dry conditions (2015–2016), and post-dry period (post 2017–2018) were developed as predictors (Table 3).

**Table 3.** Comparative fit of alternative models relating to rabies cases during the period pre-dry conditions, dry conditions and post dry conditions

	Predictors	AIC
Pre-dry period	*Vegetation index (July 2013)	1462
	*Vegetation index (May 2013)	1473
	Average temperature (Dry season 2012)	8091
	Average vegetation index (dry season 2010)	8145
	Average vegetation index (Dry season 2009)	8217
	Average vegetation index (dry season 2011)	8216
Dry period	*Average temperature (May 2015) + Vegetation index (May 2015)	1305
	*Annual average temperature 2015 + Vegetation index (Jan 2015)	1395
	*Annual average temperature 2016 + Vegetation index (June 2016)	1438
	*Annual average temperature 2015 + Vegetation index (July 2015)	1440
	2015.avtemp + Vegetation index (May 2015)	1752
	*Average temperature (Oct 2016) + Vegetation index (Oct 2016)	1752
Post dry-period	*Rainfall total (dry season) + Average temperature (June)	1294
	*Average temperature (Aug) + Vegetation index (May)	1305
	*Average temp (Sept) + vegetation (May)	1306
	*Rainfall total (dry season) + Average temperature (dry season)	1308
	*Average temperature (dry season)	1308

Models with good fit/low AIC values have an asterisk symbol (*). Models on top per each period are the most parsimonious.

### Vegetation index data

The normalised vegetation index difference for the selected periods**,** pre-dry conditions (2009 to 2013/2014), during dry conditions (2015–2016), and post-dry period (post 2017–2018) were retrieved from PROBA V data. *PROBA-V* is a small satellite assuring the succession of the Vegetation instruments on board the French SPOT-4 and SPOT-5 Earth observation missions. The data were downloaded**,** missing data pixels removed, georeferenced and area of interest clipped using the Gauteng Province shapefile. The annual maximum, minimum and mean NDVI values were calculated for selected periods**.** Furthermore, monthly dry season values (May–October) were also derived.

### Model fitting

We modelled the presence/absence of rabies cases as a function of environmental predictors using logistic regression [31]. For fitting all models, backward stepwise procedures in R software and the Akaike Information Criterion (AIC) were applied to assess the relative fit (R version 4.0.2 (2020-06-22) – ‘Spring Dance’ Copyright (C) 2020. The R Foundation for Statistical Computing). AIC model selection procedures provided the relative support for each individual model via comparing AIC values between the various models [32]. Burnham and Anderson [31] proposed that models with delta AIC values <2 have equally good fit and in the event of several models presenting delta AIC values of <2, the model with the fewest parameters (i.e. the most parsimonious) is the best [32]. Models with substantial support that **c**ould be considered candidates for the best model should be within 4–7 AIC units of the best model**,** and models with delta AIC values >10 are not supported.

## Results

### Primary rabies diagnosis

**Fig. 2.**
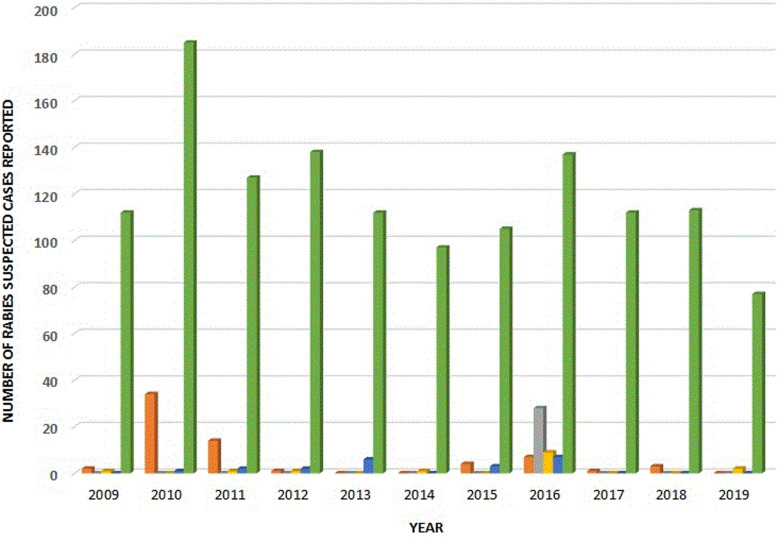
Rabies trends in Gauteng, 2009–2019.

The majority of the specimens, 72.1% (*n* = 137) were submitted as intact brain tissues in glycerol-saline preservative, and 27.9% (*n* = 53) were submitted as carcasses. Two jackal specimens (695/16 & 848/16) were deemed unfit for testing. Of the 190 specimens received, 27.9% were positive for lyssavirus antigen (*n* = 52) and 71.6% (*n* = 136) were negative. More than half of the 52 positive samples (*n* = 28, 53.8%) were recovered from black-backed jackals**.** This is the highest incidence observed in this wildlife carnivore from Gauteng Province between 2010 and 2020. With the exception of two bovine samples (592/16 & 988/16), both with a + 3 grading, apple-green fluorescing viral particles typical of lyssavirus infection against a reddish background were observed in 100% of the microscopic fields of all the smears examined under UV-fluorescence**.** Trend analyses showed that lyssavirus antigen was laboratory-confirmed consistently in domestic dogs throughout the 10 years with up to 40 infected dogs observed during the 2010 & 2011 outbreak, and fewer than 10 infected dogs observed for each of the years from 2015 to 2018 (Fig. 2). However, many RABV positive cases were laboratory-confirmed in black-backed jackals in 2016, coinciding with the 2016 rabies outbreak in this wildlife species on the periphery of the province.

### Antigenic typing

The canid RABV biotype isolates gave two different reactivity patterns, with differences in reactivity observed only with a single mAb**,** 32GD12 (Table 2). The majority of the jackal viruses (*n* = 24, 85.7%) were stained by this mAb (referred to as reactivity pattern I). Although viruses that conformed to canid biotype reactivity pattern II were less common (usually seen to react with viruses from canids from Brits and Thabazimbi located in the north-west region of South Africa, unpublished data), they exhibited a similar geographic distribution to viruses that conformed to canid biotype reactivity pattern I (Fig. 1). Spillover of infection from jackals to dogs as well as jackals to cattle was evident.

### Total RNA extractions, RT-PCR, nucleotide sequencing and phylogenetic reconstruction

**Fig. 3.**
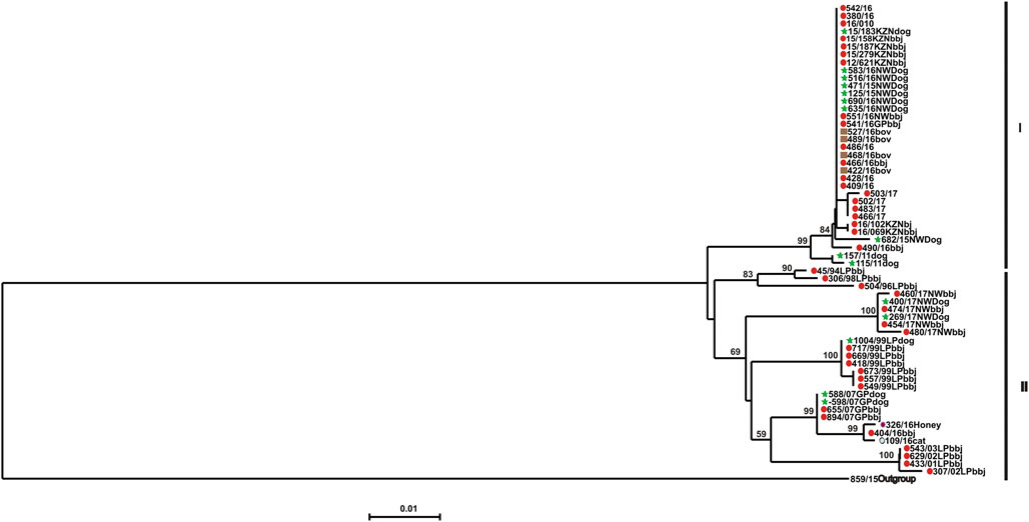
Phylogenetic analysis of RABVs included in this investigation. A 592-nucleotide portion encompassing the cytoplasmic domain of the glycoprotein and the G-L intergenic regions of the virus isolates included in this study. A neighbour-joining tree of the G-L intergenic region sequences illustrating the genetic relationships of canid RABVs from Gauteng, Limpopo, North West and KwaZulu-Natal provinces respectively. The viral sequences obtained from both black-backed jackal and domestic dogs from KwaZulu-Natal are in black, Gauteng viral isolates are in red, North West province in green and Limpopo province in dark blue. The horizontal lines are proportional to the evolutionary distances between sequences and the scale bar represents nucleotide substitutions per site. Key: bbj, black-backed jackal; dog, domestic dog; bov, bovine; cat, wild cat

Phylogenetic reconstruction using nucleotide sequences of a 592-nucleotide region encompassing the cytoplasmic domain of the glycoprotein and the G-L intergenic regions of the RABVs from the 2016 outbreak and other historically sequenced RABVs demonstrated two very distinct clusters, and the nodes supporting these clusters had significantly high bootstrap values of 84% and 83% respectively. One cluster was composed exclusively of jackal RABVs (canid rabies biotype) from the 2016 outbreak as well as other viruses from the wildlife host carnivores from the North-West province**.** Interestingly RABVs recovered from the 2012 jackal rabies outbreak in KZN, were 97.7% nucleotide sequence identical to those from the 2016 Gauteng outbreak probably demonstrating a common progenitor. The second cluster consisted of viruses originating from wildlife (jackals, honey-badgers and a wild-cat) (sub-cluster 1 of the recent RABVs) and the other sub-cluster consisted of dog and jackal viruses (mean sequence identity of 99.6%) (Fig. 3). The topology of both the NJ and ML trees were similar (only the NJ tree is shown).

### Rabies cases and environmental covariates

Table 3 presents models per period**:** pre-dry conditions, during dry conditions and post-dry conditions. Models with low AIC have an asterisk symbol (*) (i.e. AIC values <2 of best model) demonstrating evidence of good fit. Models on top per each period are the most parsimonious.

Evidence suggests that prior to the dry period of 2015–2016, although forage production was relatively high across the Gauteng Province, RABV cases were not associated with vegetation indices. Prior to the dry period (2015/2016), all models of RABV cases as a function of vegetation indices, temperature or total rainfall exhibited a poor fit (i.e. not significant) implicating other factors influencing rabies incidences. Further evidence suggests that the onset of the dry period was progressive from around 2013 onwards. After 2013, a weak but significant association of rabies cases with vegetation was becoming evident (Table 3). This relationship continued to strengthen during the drought and became even stronger after the drought (Table 3). Only models for the dry season during the drought period and post-drought period somewhat received substantial support and were closer to the best model.

## Discussion

We undertook this study to establish the origin of the 2016 rabies outbreak that occurred in jackal species in Gauteng Province and to identify the possible drivers resulting in the expansion of the range of this wildlife host species. It had always been thought that Gauteng Province was a rabies-free province. However, the dog rabies outbreak in 2010 [33], representing a major outbreak arising from an imported RABV-infected dog from KZN, and more recently, the jackal rabies outbreak on the periphery of the province in 2016 (this study), dispute the notion of a rabies-free Gauteng Province. Furthermore, these outbreaks (2010, 2012 and 2016) highlight that rabies can emerge and re-emerge should environmental conditions become conducive or in areas where it has been previously under control.

Investigations of the two rabies outbreaks in Gauteng show that from the beginning of 2010 to the end of 2011, 16.5% of the specimens tested at ARC-OVI were lyssavirus positive, with the majority of the RABVs being of dog origin. One human fatality to rabies infection was reported during the 2010 outbreak and the source was traced to a dog rabies cycle from KZN [33]. Jackal rabies has historically been associated with the northern Limpopo Province and the North-West Province (Fig. 1). In 2016, jackals were observed on the periphery of Gauteng Province and characteristically displayed loss of fear for humans, a typical symptom displayed by rabid wildlife species. Jackal rabies cases were laboratory-confirmed in 53.8% of the samples tested from Gauteng in 2016. The large proportion of rabies positive cases in jackals coupled to an unprecedented dry period in the country led us to explore the exact drivers for the movement of this wildlife host species from its historical niche areas into Gauteng Province [34, 35].

The nucleoprotein (N) is the most abundant protein produced during lyssavirus replication, making it a useful target for the diagnosis of lyssaviruses and for the differentiation of lyssavirus variants. For instance, monoclonal antibody typing detects nucleocapsid epitopes that are characteristic of particular lyssavirus species/variants (Table 2). Hence, a specific reactivity pattern defines the biotype. These antigenic types demonstrate specific events or situations including spill-over of infection and reservoir host species of a specific biotype.

In South Africa**,** dogs, bat-eared foxes and black-backed jackals maintain the canid rabies biotype within specific geographical zones, and consistently give homogeneous reactivity patterns different to those of the mongoose rabies biotype on antigenic typing. Previously, it was observed that canid RABVs produced two reactivity patterns and these differed by a single mAb (32GD12) [27] (Table 2). The epitope recognised by mAb 32GD12 is now believed to be unstable given that after the passage of some RABVs in neuroblastoma cells (C-1300), reactivity with mAb 32GD12 was restored suggesting that the epitope recognised by this mAb is indeed unstable.

Reverse-transcription polymerase chain reaction (RT-PCR), sequencing of specific gene products and reconstruction of phylogenetic trees that demonstrate genetic relationships between taxa**,** are now more widely accepted for the diagnosis of rabies particularly in laboratories adequately equipped for applying molecular technologies. These technologies underpin lyssavirus surveillance further contribute to risk analysis and improve knowledge and understanding of disease epidemiology. Three regions of the rabies viral genome, the N, the P and the G-L intergenic gene regions, are generally sequenced for inferring molecular relationships amongst lyssaviruses. The N gene is highly conserved, a feature that makes it useful for long-term evolutionary studies. For studies more focused towards a better understanding of the spread of a specific lyssavirus variant in a much smaller geographical unit, less conserved and highly variable genomic regions such as P or the cytoplasmic domain of the glycoprotein and the G-L intergenic regions, are more appropriate as targets for short-term genetic evolutionary events [27].

Genetic typing by nucleotide sequence analysis is more precise than Mab typing as it facilitates the determination of the origin of an outbreak/case. Phylogenetic analysis using sequences from our existing RABV nucleotide sequence database discriminated the viruses involved in the two rabies outbreaks of 2007 and 2016. It is evident that RABV infection was introduced into Gauteng Province via two independent albeit unrelated introductions, one in 2007 (in the north of the province bordering the Limpopo Province) and the most recent in 2016 in the western part of the province. The rabies outbreak in 2007 was probably initiated by a dog-RABV variant, whereas the 2016 rabies outbreak involved an expansion of a jackal rabies cycle from the North-West Province with spillover into dogs and cattle. Interestingly, the jackal viruses involved in the recent Gauteng Province outbreak (in 2016) were closely linked (genetically) to those from the 2012 jackal outbreak in KZN, a province that has been associated with dog rabies for over four decades [36]. The domestic dog is the principal vector species maintaining rabies transmission cycles in KZN [35, 37, 38]. However, in 2012 a rabies outbreak in the black-backed jackal population in the uThukela area of KZN is believed to have resulted from CSTEs. This is the first time that a wildlife host species has sustained a rabies outbreak independent of domestic dogs in this part of the country. Confirmed rabies cases in South Africa suggest that jackals have either increased in numbers or have expanded home ranges and this may partly explain the emergence of jackal rabies outbreaks in KZN and Gauteng Province, in 2012 and 2016. Coetzer *et al*., 2017 [39] linked the 2012 jackal rabies outbreak in KZN to an endemic sylvatic cycle. However, an independent research group attributed jackals to be the progenitor of the 2012 rabies outbreak [40]. Our data analyses confirm a close genetic link between the 2012 KZN rabies outbreak and the jackal rabies cycle from the North West Province in 2016. It was observed during the 2012 jackal rabies outbreak in KZN that 62% of the positive rabies cases originated from this wildlife host species, and that overall dog rabies cases had been reduced by 93% through parenteral dog vaccination (Department of Agriculture, Land Reform and Rural Development, KZN). Rabies outbreaks in black-backed jackals generally followed outbreaks in domestic dog populations in Zimbabwe, highlighting dogs as the potential source of the disease in wildlife [18], contrary to events leading to the KZN 2012 jackal outbreak. Contact between wild carnivore species such as jackals with domestic dogs is a common observation in Zimbabwe and this physical contact provides an ideal opportunity for rabies transmission [14, 18, 41], given the highly opportunistic nature of the canid rabies variant [11, 26].

Spatio-temporal analyses can detect clusters of infectious disease spread and are pivotal in enhancing our understanding of the dynamics of disease dispersion including dispersion of rabies. Analysis of spatial and temporal variations facilitate the identification of high-risk localities and periods of higher transmission risks, and are key attributes in surveillance and control of infectious diseases. In the case of South Africa, spatial and temporal analyses can inform policy makers on appropriate strategies for disease control and management [42]. In the past, methods were developed to model some of the aspects of infectious diseases in human populations and to some degree in companion and food animals [29, 30, 42], but is difficult to quantify the space-time epidemic processes for those diseases vectored by wildlife carnivore species [42].

In many parts of the world, wildlife diseases such as rabies are not regularly monitored**.** Often times when they are studied, the infection rates can be heterogeneous over different landscapes in addition to confounding by host factors such as age, sex and genotype [41, 43]. This requires the infection data to be analysed using statistical methods that can account for spatial, temporal and host-level heterogeneities. One limitation with reference to rabies in *C. mesomelas* is the general lack of active surveillance data for wildlife. Sampling efforts to accurately quantify rabies in jackal species in South Africa are not available despite adequate evidence for the presence of rabies in this host species in Limpopo and the North West provinces [5, 12]. A different source for disease prevalence data is veterinary diagnostic laboratories (Onderstepoort Veterinary Institute and the Allerton Provincial Laboratories), where routine rabies testing is performed. The data collected from national surveillance systems for rabies come with inherent limitations including underreporting due to inadequate surveillance. Passive surveillance has two main drawbacks as it makes the findings reported here only directly applicable to a sub-population of jackal species. The majority of jackal specimens submitted for testing likely had a history of human/domestic animal contact or loss of fear for humans, thereby introducing both a population and spatial bias. To circumvent this problem, carefully designed prospective studies to eliminate all population and spatial biases are warranted. The present study therefore underscores a need for such an effort since any and all of the information currently available on the space-time dynamics or eco-epidemiological drivers of jackal rabies exists only in the form of passive surveillance data.

The threat of rabies to communities in South Africa has led to several studies on jackal and mongoose populations [37]. Rabies transmission in mongooses, jackals and foxes are density-dependent. In addition to population density, wide-ranging movements in search of food could facilitate re-infection of terrestrial animals in rabies-free areas and equally reduce the efficiency of rabies control programmes. Black-backed jackals forage in pairs and are found throughout southern Africa where they have a wide habitat tolerance in different ecosystems including the Nama-Karoo, open savannas, arid grasslands, woodlands and deserts [38]. The species can also live in peri-urban areas, and open terrain such as farms and even suburbs**,** making Gauteng Province a potential habitat. Immature jackals (<3 years of age) move over significantly large areas, up to 30 km during a single night**,** although home ranges of 244 km^2^ during the winter months have been recorded [44].

Winkler *et al*. [35], using both drought severity and indicators of relative drought duration**,** suggested that the 2015/16 summer rainfall season in the terrestrial Southern African Development Community (SADC) region was the most severe since the droughts of the early 1980s and 1990s. In the 2015/16 rainy season, above-normal maximum temperatures, especially during early- and mid-summer, exacerbated the longer-term effect of below-normal rainfall that continued from the 2014/15 season [43]. The positive association of rabies incidences with NDVI values during the prolonged dry period (2015–2016) implies the availability of forage resources of prey species for jackals**.** This in turn suggests an indirect mechanism for food availability for rabies hosts, the yellow mongoose (*Cynictis penicillata*) and jackal species**,** as predominant factors influencing this relationship. High temperatures and low rainfall can be directly associated with accelerated drying or non-retention of greenness in forage species [45, 46], thus presenting a weak but still positive association with rabies incidences. The preliminary spatial analysis data indicated that an increase in jackal rabies cases in Gauteng Province resulted from a secondary mechanism of prey availability for the rabies hosts. When food became scarce during dry periods, jackals and mongoose**s** tended to move widely in search of prey [46, 47]. These wide movements bring them closer to human communities where virus exchanges with domestic dogs and humans can potentially occur. Territorial jackals are generally more spatially confined than dispersing jackals**,** and travel in straight lines and distances in excess of 8 km from core areas to seek food and water resources. In contrast, dispersal and floater jackals wander over long distances seeking those areas with the least competition from dominant jackals for space and food resources**.** In addition, the wandering facilitates breeding opportunities. Therefore, worsening drought conditions and associated food deprivations are pivotal factors in the poor performance of jackals during this period. The increased availability of food from the extraordinarily large amount of ungulate drought-related mortalities that were present in the field during the latter half of 2015 likely favoured jackal pup survival. The findings of this study are important in the context of rabies management in relation to wildlife (such as jackals and mongooses) in South Africa and the sub**-**region. It elucidates the contributions and relevance of extreme climatological factors as risk factors facilitating wildlife to human rabies transmissions.

The rabies outbreak in Gauteng Province in 2016 primarily involved black-backed jackals [34], a wildlife carnivore host found in peri-urban environments. The ecological conditions tended to promote an increase in jackal population numbers. Previous studies reported the highest incidence of rabies in this wildlife species during the winter months [36, 37]. During the 2016 jackal rabies outbreak, common risk contacts for potential rabies transmission included bite contact and handling of infected carcasses. Thirty-four people received post-exposure prophylaxis as they were directly exposed to the jackals and/or infected livestock, further highlighting the public health significance of rabies to farm and field workers.

The emergence of rabies outbreaks in geographical areas where rabies was previously under control, including heightened incidences in provinces where rabies is endemic**,** has raised concerns about possible problems associated with an increase in jackal populations or their movements. Provincial control efforts in response to these outbreaks and aimed at eliminating rabies in Gauteng were immediately implemented. These measures were effective in eliminating these outbreaks and are convincing as dog rabies cases in Gauteng Province decreased from 2017 onwards. Parenteral dog vaccination campaigns have led to an overall decrease in the number of dog and human rabies cases. Vaccination of domestic dogs should therefore be a priority but ideally complemented with baited vaccines to reach stray dogs and other wildlife hosts in order to eliminate dog-mediated human rabies in South Africa and the region. As demonstrated in Europe, oral rabies vaccination has been a proven and effective tool to eliminate rabies in domestic dogs. Practically, the whole of Europe is rabies-free but the risk of re-introduction of the disease always exists, through either the transboundary routes, and/or animal and human trafficking. In South Africa and probably in other regions of the continent, vaccination of domestic dogs should ideally be performed in synergy with the other wildlife carnivore species that maintain sylvatic rabies cycles.

## Conclusion

In conclusion, this study has demonstrated an expansion of a jackal rabies cycle from North West Province into the Gauteng Province during the dry period of 2016, as the black-backed jackals ranged widely in search for food resources. This resulted in jackal-dog interactions, reminiscent of the links between domestic and wildlife rabies cycles in South Africa. Clearly, the transmission of the RABV is neither restricted by administrative/provincial boundaries nor the history of occurrence of the disease, but is modulated by the surrounding environmental variables, such as climate and**/**or human habitats which in turn exhibit spatial heterogeneity. Thus, to control rabies**,** it is necessary to analyse risks associated with rabies at the case level in order to identify the influence of the geographic determinants on case distributions. The latter is crucial because the spatial pattern of human rabies cases has strong relations with the distribution and movement of rabies in animal hosts and likely the movement of stray and roaming dogs.

## Data Availability

The data used to reach conclusions in this publication are publicly available as nucleotide sequences of RABVs can be retrieved from Genbank using the specific accession numbers for the taxa included in the publication. The raw data that was used to generate rabies trends for Gauteng Province can be requested from the Agricultural Research Council (South Africa) and can be provided through either, a materials transfer agreement (MTA) or project agreement.
